# Prognostic value of subclinical myocardial necrosis using high-sensitivity cardiac troponin T in patients with prediabetes

**DOI:** 10.1186/s12933-021-01365-9

**Published:** 2021-08-21

**Authors:** Marco Witkowski, Yuping Wu, Stanley L. Hazen, W. H. Wilson Tang

**Affiliations:** 1 Department of Cardiovascular & Metabolic Sciences, Lerner Research Institute, Cleveland Clinic, Cleveland, OH USA; 2 Department of Mathematics, Cleveland State University, Cleveland, OH USA; 3 Department of Cardiovascular Medicine, Heart, Vascular and Thoracic Institute, Cleveland Clinic, 9500 Euclid Avenue, Desk J3-4, Cleveland, OH 44195 USA

**Keywords:** Prediabetes, Cardiac troponin T, Prognosis

## Abstract

**Background:**

Risk stratification of patients with prediabetes is an unmet clinical need. Here, we examine the utility of subclinical myocardial necrosis assessed by high-sensitivity cardiac troponin T (hs-cTnT) in predicting health outcomes in stable subjects with prediabetes.

**Methods:**

hs-cTnT was analyzed by a high-sensitivity assay (Roche 5th generation) in 2631 stable subjects with prediabetes (HbA1c 5.7–6.4% or fasting glucose 100–125 mg/dL without previous diagnosis of diabetes or glucose-lowering therapy) who underwent elective coronary angiography for cardiac evaluation, and followed for major adverse cardiac events (MACE; death, myocardial infarction, stroke) over 3 years and all-cause mortality over 5 years.

**Results:**

In our study cohort, hs-cTnT was highly prevalent with a median level of 13 ng/L (interquartile range 8.2–21.6 ng/L). Hs-cTnT was independently associated with incident MACE at 3 years (Q4 vs. Q1 adjusted hazard ratio (HR) 2.42 [95% CI 1.69–3.46], P < 0.001) and 5-year mortality (adjusted HR 3.8 [95% CI 2.55–5.67], P < 0.001). This association remained significant in all subsets after adjustment for traditional risk factors and multiple factors known to increase hs-cTnT levels. Moreover, hs-cTnT independently predicted event risk in primary prevention subjects (n = 557, HR 5.46 [95% CI 1.50–19.89), p < 0.01) for MACE; HR 9.53 [95% CI 2.08–43.73] for all-cause mortality) and secondary prevention subjects (n = 2074, HR 1.86 [95% CI 1.31–2.66], P < 0.001 for MACE; and 2.7 [95% CI 1.79–4.08), P < 0.001 for all-cause mortality).

**Conclusions:**

In stable prediabetic subjects, the presence of subclinical myocardial necrosis as detected by hs-cTnT portends heightened long-term adverse cardiovascular event risk. Hs-cTnT levels may help to stratify risk and improve clinical decision making in patients with prediabetes.

*Trial registration* ClinicalTrials.gov Identifier: NCT00590200.

**Supplementary Information:**

The online version contains supplementary material available at 10.1186/s12933-021-01365-9.

## Introduction

Prediabetes, defined as glycemic concentrations above normal but lower than diabetes thresholds, has emerged as a new category of abnormal glycemic control. Besides impaired fasting glucose (IFG) or impaired glucose tolerance (IGT), the American Diabetes Association recently introduced glycated hemoglobin A1c (HbA1c) 5.7–6.4% to define prediabetes [1]. Population-based survey data estimates that more than one third of the United States population (> 84 million people) can be considered “prediabetic” based on IFG or HbA1c [2]. While the well-known association between diabetes and macrovascular complications justifies aggressive medical interventions, cardiovascular risk stratification for individuals with dysglycemia in the pre-diabetic range has proven difficult and is complicated by different classifications and cutoffs for prediabetes [3]. In fact, many studies report that prediabetes does not portend the same increase in cardiovascular disease (CVD) risk as seen in diabetes and association of prediabetes with cardiovascular complications are inconsistent throughout multiple studies both in the general population [4–8] as well as secondary prevention subjects [9–11]. Therefore, a test that helps to identify those individuals that are at increased cardiac risk amongst prediabetic patients would be very helpful.

Since the availability of high-sensitivity cardiac troponin T (hs-cTnT) assays, the prognostic utility of subclinical myocardial necrosis (SMN) has gained growing appreciation in their ability to predict adverse long-term cardiovascular risk in stable cardiovascular patients [12, 13]. We and others have previously shown that hs-cTnT is associated with major adverse cardiac events (MACE) in patients with diabetes [14] and that prediabetes is independently associated with incident of SMN and CVD risk in community-based populations [6, 15]. We therefore hypothesize that SMN, as detected by hs-cTnT, may portend poor prognosis in a stable cohort of prediabetic subjects (with no clinical or laboratory evidence for an acute coronary syndrome at enrollment) undergoing elective diagnostic cardiac evaluations. Herein we examined the clinical prognostic utility of hs-cTnT in both primary and secondary prevention subjects as a biomarker to identify prediabetic subjects at increased cardiovascular risk and thus appropriate for more aggressive preventive efforts.

## Methods

### Study population

The Cleveland Clinic GeneBank Study is a large, single-center, prospective cohort study with a thoroughly characterized collection of clinical data and longitudinal outcomes in stable patients undergoing elective diagnostic angiography because of suspected CAD or progression of known CAD at the Cleveland Clinic between 2001 and 2007 (detailed inclusion and exclusion criteria can be found at ClinicalTrials.gov Identifier: NCT00590200). All participants gave written informed consent to a protocol that was approved by the Cleveland Clinic Institutional Review Board. This analysis included 2,631 stable cardiac patients without acute coronary syndrome (cardiac troponin I ≤ 0.3 ng/mL) and with a diagnosis of prediabetes, defined as no history of diabetes mellitus or use of glucose-lowering drugs, and with HbA1c levels between 5.7 and 6.4% or fasting plasma glucose levels between 100 and 125 mg/dL.

Primary prevention was defined as those without CVD, which included either coronary artery disease (CAD, any clinical history of myocardial infarction, coronary revascularization [such as percutaneous coronary intervention, coronary artery bypass surgery], or angiographic evidence of significant stenosis [≥ 50%] in 1 or more major coronary arteries) or peripheral artery disease (non-coronary arterial territories including extracranial carotid artery stenosis, upper extremity artery stenosis, renal artery stenosis, and lower extremity arterial diseases, while diseases of the aorta were not included). Secondary prevention was defined as those with evidence for CVD. Estimated glomerular filtration rate (eGFR) was calculated via CKD-EPI equation [16]. Subjects were followed for 3 years following enrollment to determine the occurrence of MACE, defined as all-cause mortality, nonfatal myocardial infarction, or nonfatal stroke. Subjects were also followed for 5 years for all-cause mortality. All endpoints in our cohort were collected by in-person prospective follow-up including letter solicitation and reply cards, chart review, and direct contact by study staff by follow-up telephone interviews, and were adjudicated and confirmed by source documentation over the ensuing 3 years after enrollment. All-cause mortality was determined by chart review confirmed by Social Security Death Index 5 years after enrollment.

### Subclinical myocardial necrosis

During the time of the cardiac catheterization procedure, all blood samples were collected after arterial sheath access had been obtained and prior to diagnostic catheterization or treatment, including heparin administration. High-sensitivity cardiac troponin T levels were measured on a Roche Cobas E411 with 5th generation research assay (Roche laboratories, Indianapolis IN) at CAP/CLIA-approved research laboratory. The limit of detection was 3 ng/L and there were no values measured below this level in this cohort. The 99th percentile cutoff was 14 ng/L with an average coefficient of variation < 10% at that level. High-sensitivity C-reactive protein (hsCRP) and lipid profiles were also measured by the same platform.

### Statistical analysis

Continuous variables were summarized as mean ± standard deviation if normally distributed and median with interquartile range [IQR] if non-normally distributed. Cox proportional hazard analysis was used to assess the clinical risks associated with hs-cTnT levels as stratified in group quartiles (Q). Adjustments were made for age, gender, systolic blood pressure, low-density lipoprotein cholesterol, high-density lipoprotein cholesterol, former/current cigarette smoking and eGFR to predict the 3-year MACE risks or all-cause mortality at 5 years. In additional analyses, we included CVD, hsCRP, HbA1c or left ventricular ejection fraction (LVEF) in addition to the above-mentioned risk factors into the adjustment. We confirmed that both the proportionality hazards and linearity assumptions were met. We used Cochran-Armitage and Jonckheere–Terpstra tests of trend to compare baseline characteristics across increasing quartiles of hs-cTnT for categorical and continuous variables, respectively. Analyses were performed with R version 4.0.2 and P < 0.05 was considered statistically significant.

## Results

Baseline characteristics of the study population are shown in Table 1. As expected, the majority of subjects (71%) demonstrated evidence of clinically significant coronary artery disease (≥ 50% stenosis at any vessel, with increasing prevalence across hs-cTnT quartiles, see Additional file 1: Table S1), with 15.5% subsequently underwent coronary revascularization within 30 days following coronary angiography. The average estimated glomerular filtration rate (eGFR) was 83.8 mL/min/1.73 m^2^. In this patient cohort without history or evidence of acute coronary syndrome, hs-cTnT was detectable in all subjects with a median hs-cTnT level of 13 ng/L (IQR 8.2, 21.6 ng/L).

**Table 1:** Baseline characteristics of study population

Variables	All subjects (n = 2631)	Quartile 1 (n = 658)	Quartile 2 (n = 653)	Quartile 3 (n = 661)	Quartile 4 (n = 659)	P value for trend
hs-cTnT range (ng/L)		< 8.2	8.2–13	13–21.6	≥ 21.6	
Age (years)	65 ± 11.2	59.9 ± 10	63.4 ± 10.5	68.3 ± 10	68.4 ± 11.9	< 0.001
Male (%)	72.4	66.3	75.2	75.2	73.1	0.001
BMI (kg/m^2^)	28.4 (25.4–31.9)	28.6 (25.8–32.7)	28.7 (25.8–32)	28.1 (25.4–31.3)	27.7 (24.9–31.6)	0.001
HbA1c (%)	5.8 (5.5–6.0)	5.7 (5.5–6.0)	5.8 (5.5–6.0)	5.8(5.4–6.0)	5.8(5.5–6.0)	0.094
(mmol/mol)	39.9 (36.6–42.1)	38.8 (36.6–42.1)	39.9 (36.6–42.1)	39.9 (35.5–42.1)	39.9 (36.6–42.1)	
Fasting blood glucose (mg/dL)	103.2 (95.2–110)	103 (94.9–110)	102.9 (94.7–110)	103.6 (97.2–110)	103.8 (95.1–110)	0.438
Risk factors						
Hypertension (%)	71	64	68	76	77	< 0.001
Former/current smokers (%)	67	65	70	67	66.5	0.394
CVD (%)	78.8	67.6	72.9	84.3	90.4	< 0.001
CAD (%)	74.8	63.1	70	78.3	87.8	< 0.001
PAD (%)	24.7	17.2	19.3	32.1	30	< 0.001
Laboratory data						
LDLc (mg/dL)	97 (79–119)	100 (82–122)	98 (81–120)	95.5 (77–116)	96 (79–117)	0.032
HDLc (mg/dL)	37 (31–46)	37 (31–46)	37 (31–45)	38 (31–47)	36 (30–45)	0.106
TG (mg/dL)	118 (85–169)	124 (86–179)	117 (87–172)	111 (83–157)	121 (88–173)	0.004
hsCRP (mg/L)	2.3 (1.0–5.3)	1.8 (0.8–3.9)	1.9 (0.9–3.6)	2.0 (0.9–4.4)	5.3 (1.9–13.5)	< 0.001
eGFR (mL/min per 1.73 m^2^)	83.7 (68.6–94.3)	90.3 (79.7–98.4)	85.2 (73.7–95.6)	78.9 (65.0–91.0)	76.2 (58.5–91.0)	< 0.001
Medications						
ACEi/ARB (%)	46.6	37.5	46.4	50.5	52	< 0.001
β-Blockers (%)	60.6	58.7	61.7	62.5	59.6	0.456
Statins (%)	57.7	55.9	60	58.5	56.3	0.388
Aspirin (%)	73	74.8	74.1	72.3	70.9	0.37

Continuous data are presented as mean ± standard deviation or median (interquartile range), categorical variables are presented as %

*ACEi* angiotensin-converting enzyme inhibitor, *ARB* angiotensin receptor blockers, *BMI* body mass index, *CAD* coronary artery disease, *PAD* peripheral artery disease, *eGFR* estimated glomerular filtration rate, *HbA1c* glycated hemoglobin, *HDLc* high-density lipoprotein cholesterol, *hsCRP* high-sensitivity C-reactive protein, *hs-cTnT* high-sensitivity cardiac troponin T, *LDL* low-density lipoprotein cholesterol, *MACE* major adverse cardiac events, *TG* triglycerides

### Subclinical myocardial necrosis and major adverse cardiac event risk among prediabetic patients

**Fig. 1:**
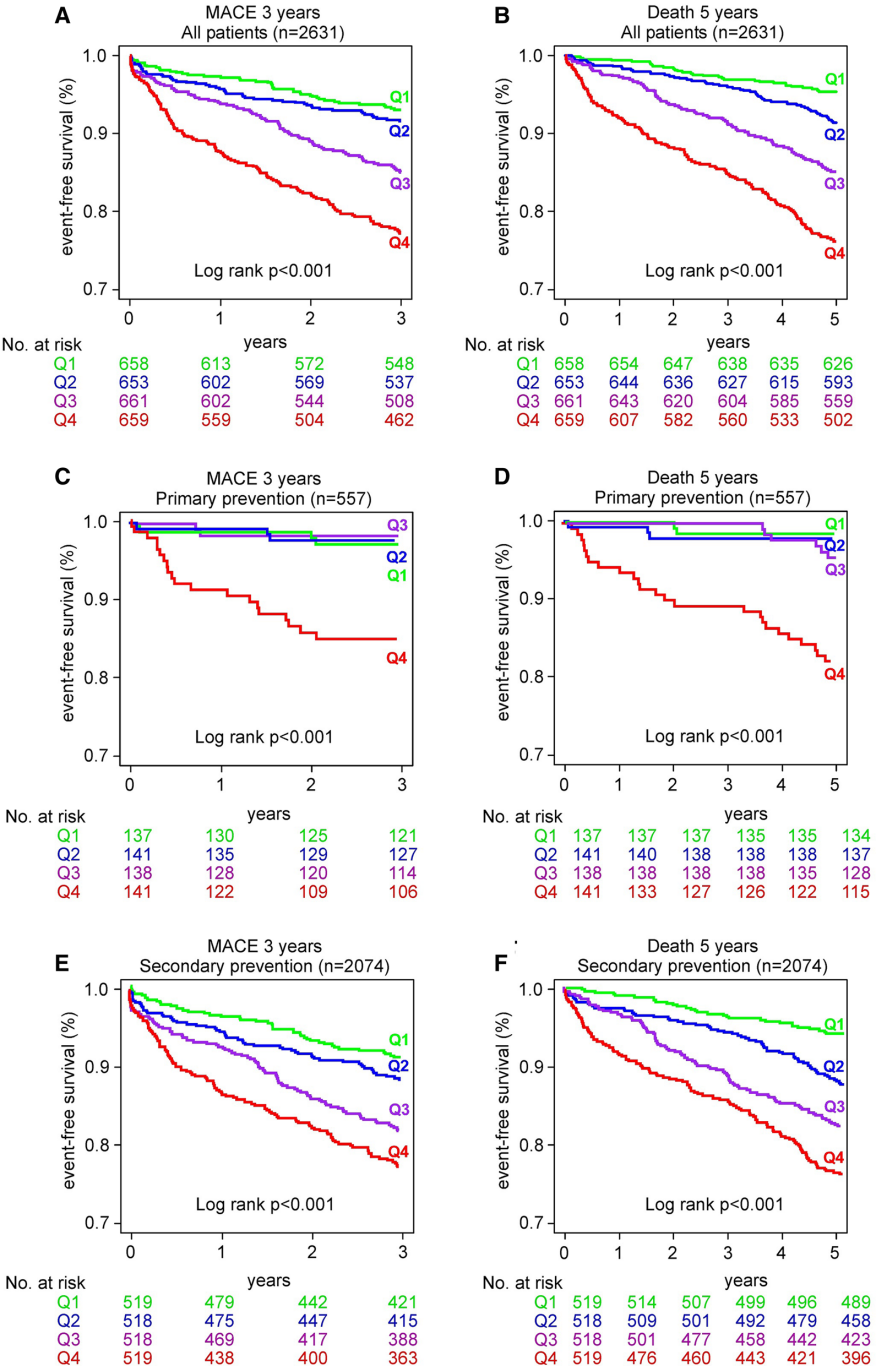
Kaplan–Meier estimates and the risk of incident major adverse cardiac events (MACE, defined as myocardial infarction, stroke, or death) as well as all-cause mortality over follow-up periods of 3 and 5 years, respectively, stratified by quartile of hs-cTnT levels. Shown is the analysis for all patients (**A**, **B**), primary prevention subjects (**C**, **D**) and secondary prevention subjects (**E**, **F**)

Figure 1 shows the Kaplan–Meier analysis for event-free survival in patients stratified by hs-cTnT quartiles. Increasing hs-cTnT levels were associated with an incremental increase in event risk for 3-year MACE and 5-year all-cause mortality in the entire cohort (Fig. 1A, B, log rank P < 0.001) especially when raised above median level (Q2).

In the subset with primary prevention subjects, those in the highest hs-cTnT (≥ 14.4 ng/L) also had a significant higher risk for 3-year MACE and 5-year all-cause mortality (Fig. 1C, D, log rank P < 0.001), while in secondary prevention patients, hs-cTnT levels above median levels (≥ 14.2 ng/L) portended an incremental risk for 3-year MACE and 5-year all-cause mortality (Fig. 1E, F, log rank P < 0.001).

**Fig. 2:**
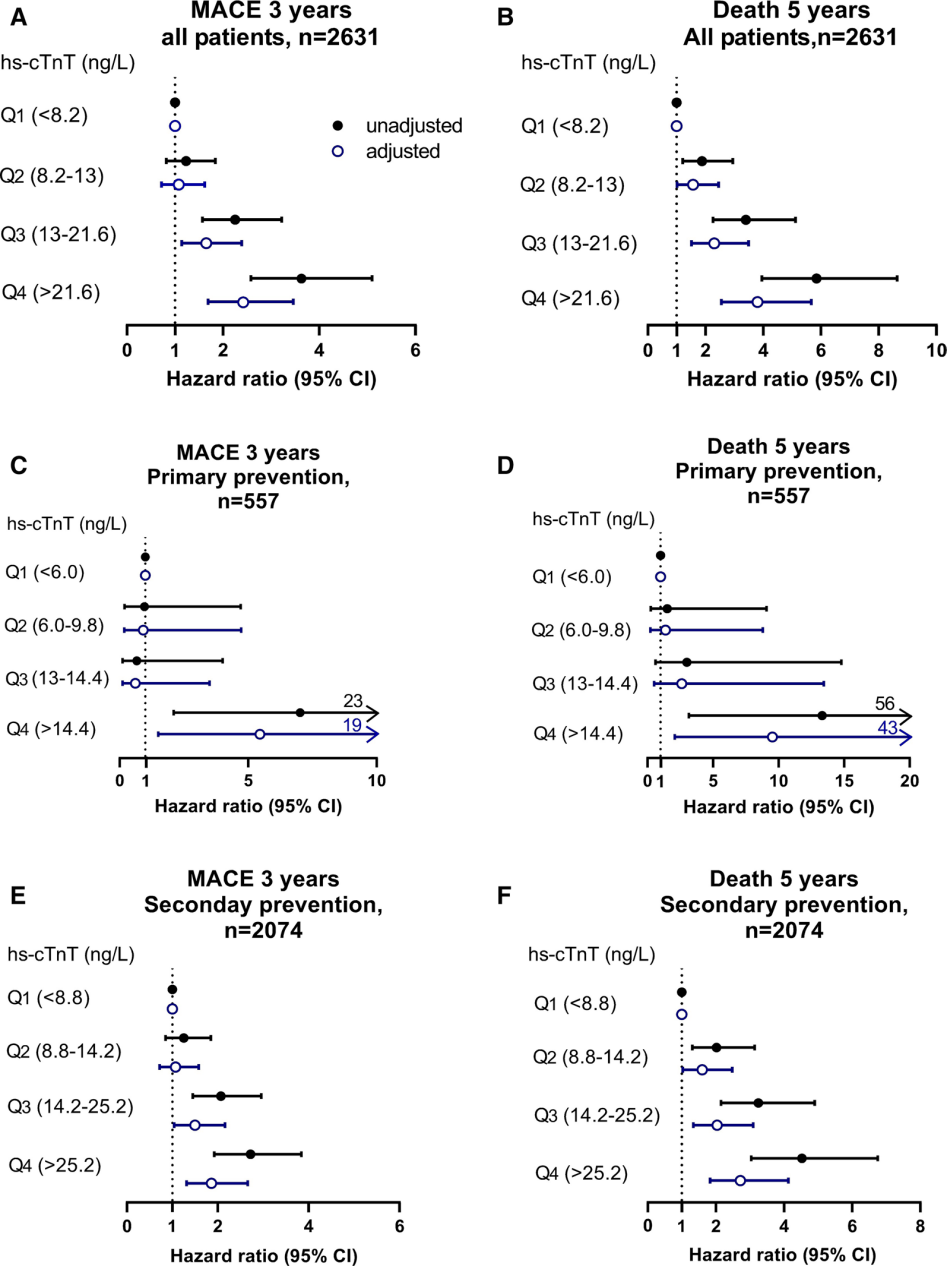
Forest plots indicating the risks of incident MACE at 3 years and death after 5 years ranked by quartiles of hs-cTnT levels. The multivariable Cox model for hazard ratio included adjustments for age, sex, systolic blood pressure, low density lipoprotein cholesterol, high density lipoprotein cholesterol, smoking and estimated glomerular filtration rate. The 5–95% confidence interval is indicated by line length. Shown is the analysis for all patients (**A**, **B**), primary prevention subjects (**C**, **D**) and secondary prevention subjects (**E**, **F**)

Figure 2 demonstrates unadjusted and adjusted Cox proportional hazard ratios (HR) for incident development of MACE in 3 years. Prediabetic patients with hs-cTnT levels in Quartile 3 (hs-cTnT 13–21.6 ng/L) and Quartile 4 (hs-cTnT ≥ 21.6 ng/L) had a 2.25-fold (HR 2.25 [95% CI 1.57–3.22), P < 0.001]) and 3.63-fold (HR 3.63 [95% CI 2.58–5.10], P < 0.001) increased risk for MACE, respectively. These increased risks for MACE remained significant after adjusting for traditional risk factors (Q3 vs. Q1: adjusted HR 1.65 [95% CI 1.14–2.39], P < 0.01; Q4 vs. Q1: adjusted HR 2.42 (1.69–3.46), P < 0.001) (Fig. 2A). In the subset analyses, higher hs-cTnT levels were associated with a fivefold increase in MACE (Q4 vs Q1: adjusted HR 5.46 [95% 1.50–19.89], P < 0.01) in the primary prevention cohort (Fig. 2C) and a 1.9-fold increase in MACE (Q4 vs. Q1: adjusted HR 1.86 [95% CI 1.31–2.66], P < 0.001, Fig. 2E) in the secondary prevention cohort following adjustments for traditional risk factors.

### Subclinical myocardial necrosis and mortality risk among prediabetic patients

Prediabetic patients with elevated hs-cTnT levels had higher 5-year mortality (Q2 vs. Q1: HR 1.88 [95% CI 1.21–2.94]; P < 0.01; Q3 vs. Q1: HR 3.40 [95% CI 2.26–5.12]; P < 0.001; Q4 vs. Q1: HR 5.85 [95% CI 3.95–8.64], P < 0.001). This association remained significant after adjustments for traditional risk factors: Q3 vs Q1: adjusted HR 2.19 [95% CI 1.44–3.34], P < 0.001; Q4 vs. Q1: adjusted HR 3.37 [95% CI 2.25–5.05], P < 0.001) (Fig. 2B). Heightened mortality risk remained significant after adjustment in the primary prevention cohort (Q4 vs. Q1: adjusted HR 9.53 [95% CI 2.08–43.73], P < 0.01, Fig. 2D) and secondary prevention cohort (Q4 vs. Q1: adjusted HR 2.70 [95% CI 1.79–4.08], P < 0.001, Fig. 2F).

When further adjusting for hsCRP, use of statins or angiotensin converting enzyme inhibitors, HbA1c or LVEF, higher hs-cTnT levels remained significantly associated with increased 3-year MACE and 5-year death in the entire cohort, as well as primary (Q4 vs. Q1–3) and secondary prevention subjects (Additional file 1: Tables S2–S4). We also performed additional analysis in the subset of patients who did not undergo coronary revascularization within 30 days following angiography, and observed similar trends, even when adjusting for traditional and above-mentioned CVD risk factors as well as degree and extent of disease burden (Additional file 1: Table S5). These sensitivity analyses highlight the independent prognostic value of hs-cTnT in this prediabetic population.

### Event risk among different subgroups

**Fig. 3:**
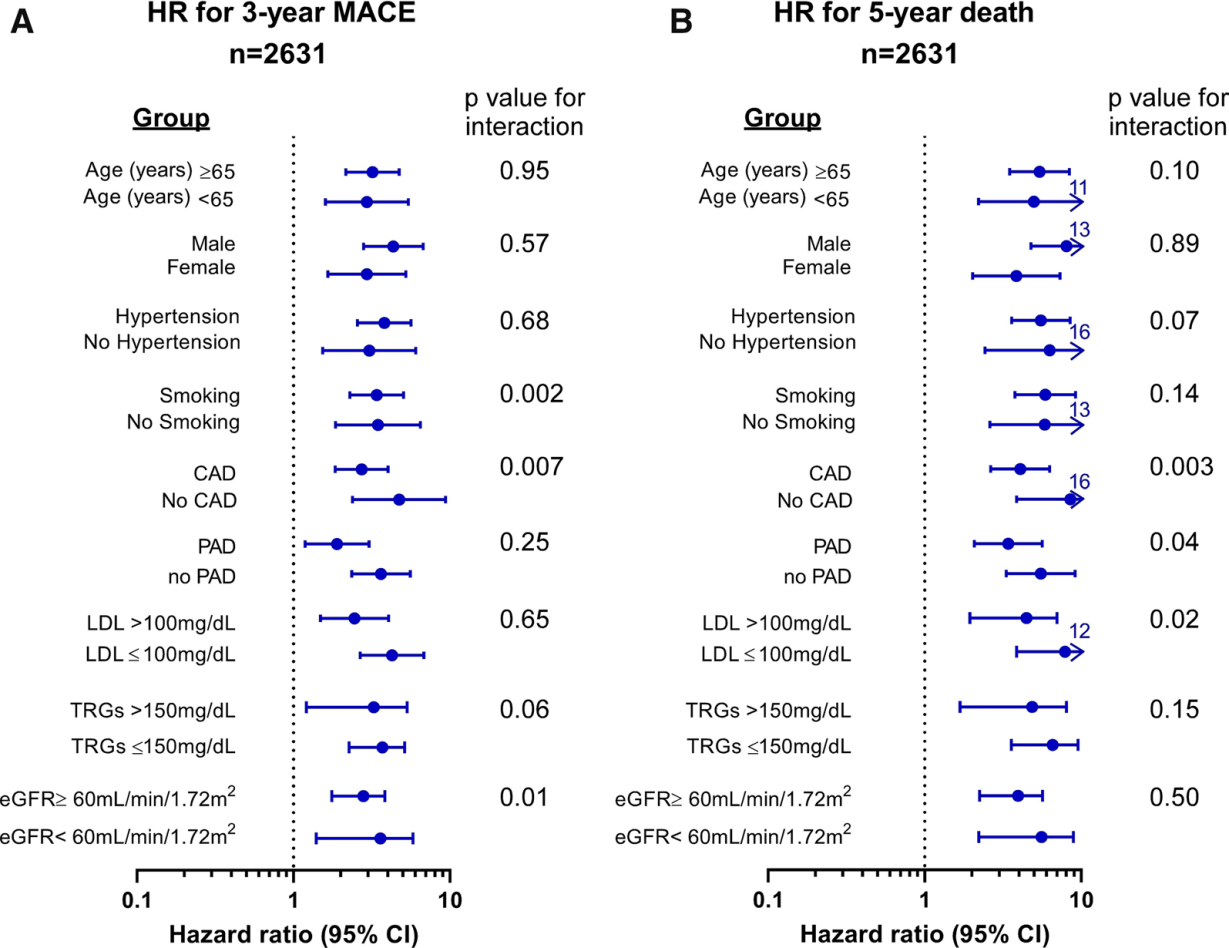
Forest plot of the hazard ratio for **A** MACE in 3 years and **B** 5-year all-cause mortality risk comparing first and fourth quartiles of hs-cTnT levels in different groups as indicated. The 5–95% confidence interval is indicated by line length. P value for trend was calculated by Cochran–Armitage and Jonckheere–Terpstra tests of trend to compare baseline characteristics across increasing quartiles of hs-cTnT for categorical and continuous variables, respectively. *CAD* coronary artery disease, *eGFR* estimated glomerular filtration rate, *LDL* low density lipoprotein cholesterol, *PAD* peripheral artery disease, *TRGs* triglycerides

**Fig. 4:**
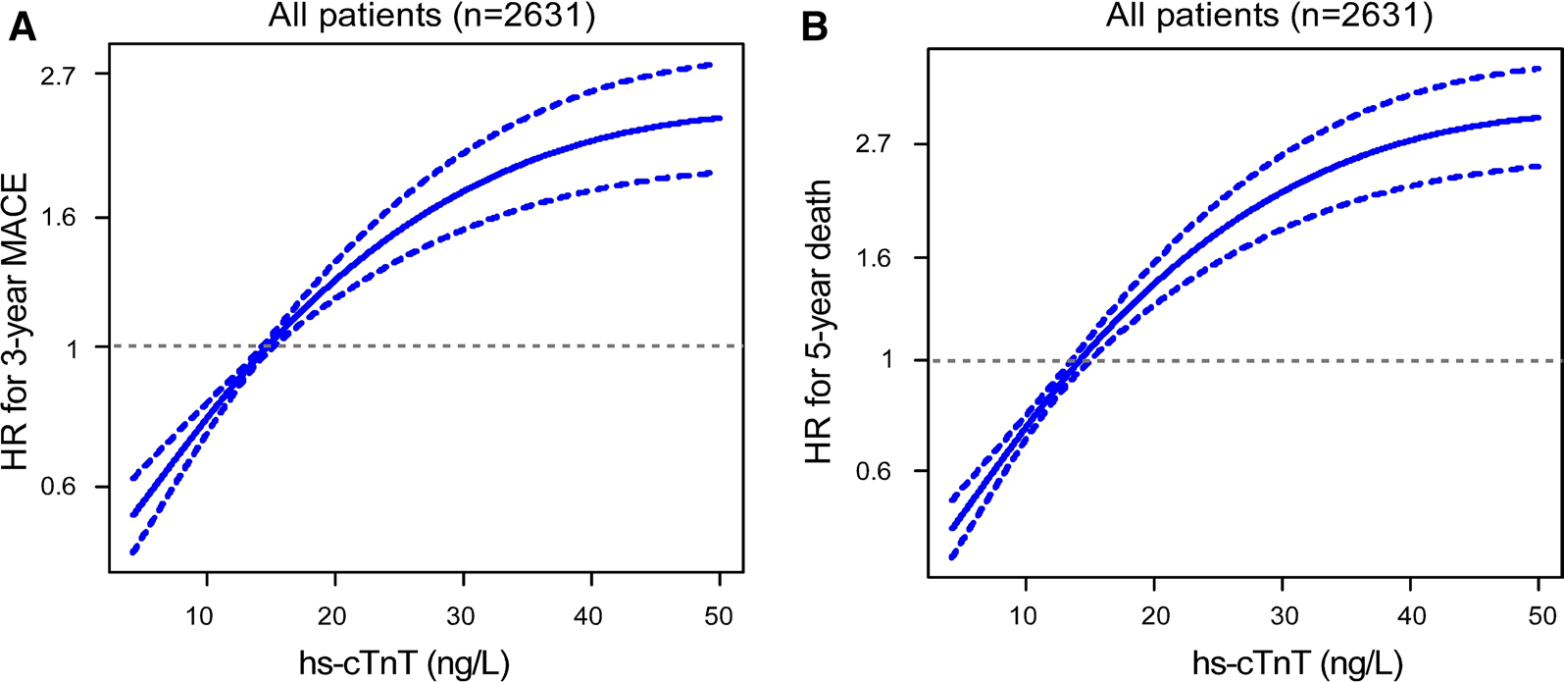
Cubic spline curves of the hazard ration (HR) for **A** major adverse cardiac events (MACE, death, nonfatal myocardial infarction, and stroke) at 3 years and **B** death at 5 years with hs-cTnT levels

Adverse event and mortality risks were similar among multiple clinical subgroups in the entire cohort (Fig. 3) including preserved and impaired kidney function. Using hs-cTnT as a continuous variable, cubic spline analyses showed a virtually linear increase in HR for MACE and 5-year all-cause mortality as hs-cTnT rises in the prediabetic population (Fig. 4). Interestingly, there was no correlation between the levels of hs-cTnT and either fasting glucose or HbA1c in our cohort (r = 0.03, P = 0.191 and r = 0.03, P = 0.146, respectively, for Spearman correlation).

## Discussion

We presented one of the largest reported cohort of patients with prediabetes undergoing elective diagnostic invasive cardiac evaluation stratified by SMN to assess cardiovascular outcomes. Plasma levels of hs-cTnT were incrementally associated with the incident risk for MACE as well as all-cause mortality after extensive multivariate adjustment for CVD risk factors and conditions known to increase plasma hs-cTnT levels. Notably, in both primary prevention and secondary prevention subjects, hs-cTnT provided significant prognostic value. Our study demonstrates that SMN is prevalent in patients with prediabetes undergoing elective diagnostic cardiac evaluation, with almost half of our study cohort above the 99th upper limit of normal. Our findings imply that quantification of plasma hs-cTnT may convey additional prognostic value for the risk stratification of individuals with prediabetes in the presence of cardiovascular risk factors.

There is ample evidence that diabetes confers CVD event risk through factors involving glycemic variability [17], inflammation [18–20], platelet dysfunction [21, 22] and a pro-thrombotic state [23–25]. Yet, the picture in the prediabetic range is less clear. While some previous studies in the general population provided evidence that prediabetes defined by fasting glucose, HbA1c or oral glucose tolerance test (OGTT) may be associated with higher risk for CVD and mortality [26, 27] many other studies did not report an association between prediabetes and CVD complications [4, 5, 7]. Risk stratification of these subjects is critical to prevent cardiovascular complications. However, the inconsistent association of prediabetes with cardiovascular events sustains an ongoing debate about prediabetes being a “dubious diagnosis” that results in unnecessary medical interventions and stresses the healthcare system [28, 29]. Our study provides evidence that extend of SMN represents a biomarker that is useful to identify prediabetic patients at risk to avoid unnecessary care and improve outcomes. Although the consequence of myocardial necrosis can be determined via other imaging-based techniques such as late gadolinium enhancement by cardiac magnetic resonance for myocardial fibrosis [30, 31], measurement of hs-cTnT is readily performed and more broadly available what makes it a more attractive diagnostic tool.

Once CVD is present in a prediabetic environment, using prediabetes as a stand-alone prognostic marker for CVD risk yielded conflicting results in stable [10, 11] or acute [32, 33] patients and in this setting prediabetes status alone fails to universally predict outcomes. On one hand, this might be explained by clustering of prediabetes state with other glycemic CVD risk factors [4, 34]. On the other hand, limited data in prediabetic patients and inconsistent clinical studies that used different classifications and cutoffs for prediabetes preclude general conclusions about event risk. Yet, the majority of patients with CVD shows an abnormal glucose metabolism [35] and novel non-glycemic biomarkers are needed for risk stratification to guide secondary prevention of patients with prediabetes. Our findings suggest that assessment of hs-cTnT can help risk stratify prediabetic subjects to guide secondary preventive efforts.

Use of next generation hs-cTnT assays greatly improved the diagnosis of acute coronary syndromes, and has been increasingly available in the acute clinical setting. Interestingly, in patients in whom an acute myocardial infarction has been ruled out in a binary approach, hs-cTnT is a strong predictor for future risk of myocardial infarction and mortality, indicating prognostic value on a continuous scale [36]. Due to their higher sensitivities, the 5th generation hs-cTnT assays allow detection of trace levels of cardiac troponins even on a general population level where it was found to be associated with heart disease and mortality risk [37]. This led to exploration of hs-cTnT as a prognostic biomarker in various stable cardiovascular phenotypes, such as CVD [12], heart failure [13] or hypertrophic cardiomyopathy [38]. Our study has the potential to expand the prognostic utility of hs-cTnT from the general population [6] or individuals with diabetes [14] to stable cardiac patients with prediabetes.

Although the mechanisms leading to troponin leakage with detectable amount of hs-cTnT in the plasma are not completely understood, chronic myocardial stress, cardiomyocyte injury and isolated subendocardial ischemia have been proposed [39]. Although CVD was more prevalent with increasing levels of hs-cTnT in our patient cohort, adjustment for CVD did not abrogate the association of hs-cTnT with event risk (Additional file 1: Tables S1–S3) and even in the absence of CVD we found SMN portends event risk (Fig. 2) suggesting other mechanisms involved.

Other factors that are associated with elevated hs-cTnT levels in general populations were age, gender, low density lipoprotein cholesterol and blood pressure [40, 41], which were included into our multivariate analysis, along with other traditional CVD risk factors and kidney function. We also observed higher levels of hsCRP with increasing hs-cTnT levels. In a recent study, Lucci et al. prospectively followed 2064 patients with acute myocardial infarction and found that hsCRP levels predicted mortality and were correlated with hs-cTnI both in patients with and without diabetes [42]. Although the link between CRP and SMN in stable patients warrants further investigation adjusting for hsCRP did not change the association of hs-cTnT in our prediabetic cohort (Additional file 1: Tables S1–S3).

In a recent pooled analysis on the population level, prediabetes status was associated with a higher lifetime risk for heart failure in middle-aged white adults and black women [43]. Another study found sex-specific maladaptive left ventricular changes as glucose tolerance worsened from normal to prediabetes [44]. Hs-cTnT levels ≥ 6 ng/L were recently added to a biomarker score for heart failure risk stratification in subject with diabetes and prediabetes [45]. Interestingly, adjustment for LVEF did not change association of hs-cTnT with event risk in our cohort (Additional file 1: Tables S1–S3).

Generally, the 99th percentile of a healthy reference population defined by the manufacturer may identify individuals with elevated hs-cTnT levels. Although in our stable prediabetes cohort a myocardial infarction has been ruled out, we observed that a significant proportion of the subjects in our cohort exceeded the reported 99th percentile of 14 ng/L for our assay. Our data highlights that prediabetes in the presence of CVD risk factors is associated with robust levels of plasma hs-cTnT. However, current reference ranges for hs-cTnT have been optimized for diagnosing acute coronary syndromes and likely need to be better defined for individual populations [46].

Our data suggest that use of hs-cTnT for risk stratification in stable prediabetes subjects undergoing elective diagnostic cardiac evaluations provides an additional tool to help identify the subset of subjects who are at a “high risk equivalent”, and may thus benefit from more aggressive modifiable risk reduction strategies. Including nonglycemic biomarkers into the risk assessment for prediabetic patients with CVD risk factors may help to overcome the limitations of using prediabetes status as a standalone measure to predict CVD outcomes. Further studies to better understand the metabolic alterations that occur concurrently with changes in glycemic control that led to the classification of subjects as “prediabetic” may prove helpful in further expanding upon the preventive strategies to try and mitigate the increased risks observed with hs-cTnT elevation in this cohort.

### Study limitations

The present study has several limitations. Measurement of hs-cTnT was only performed once at time of enrolment, during angiographic evaluation of the stable cohort. Whether serial measures provide enhanced prognostic value for incident CVD risks, or would begin to correlate with glucose or HbA1C levels is unknown. We also did not include oral glucose tolerance testing, which could have caused some misclassifications of patients. It should be noted, however, that the definition of prediabetes used was in accordance with the ADA guidelines and represents the general clinical practice that prefers fasting glucose and HbA1c over the less convenient and costlier oral glucose tolerance test.

## Conclusions

Our findings provide strong evidence detectable levels of hs-cTnT, an indicator of SMN, is prevalent in patients with prediabetes undergoing elective diagnostic cardiovascular risk evaluation. Moreover, hs-cTnT levels in this cohort were independently associated with both CVD event risk, and long term (5-year) mortality risks among all subjects, as well as among the subsets with documented CVD (secondary prevention) and without CVD (primary prevention). In subjects with prediabetes and risk factors (that represent a large population), hs-cTnT may help to stratify risk and guide preventive efforts to use healthcare resources more efficiently. The strong clinical prognostic utility of hs-cTnT suggests its incorporation into current practice guidelines for prediabetic subjects undergoing cardiac evaluations should be further studied.

## Supplementary Information

**Additional file 1: Table S1.** Coronary angiography findings and rates of revascularization within 30 days following procedure across hs-cTnT quartiles. **Table S2A.** Major adverse cardiac event (MACE) risk according to quartiles of hs-cTnT with additional adjustments in the entire prediabetes cohort. **Table S2B.** All-cause mortality according to quartiles of hs-cTnT with additional adjustments in the entire prediabetes cohort. **Table S3A.** Major adverse cardiac event (MACE) risk according to quartiles of hs-cTnT with additional adjustments in secondary prevention subjects. **Table S3B.** All-cause mortality according to quartiles of hs-cTnT with additional adjustments in secondary prevention subjects. **Table S4A.** Major adverse cardiac event (MACE) risk according to pooled quartiles 1, 2 and 3 vs. quartile 4 of hs-cTnT with additional adjustments in primary prevention subjects. **Table S4B.** All-cause mortality risk according to pooled quartiles 1, 2 and 3 vs. quartile 4 of hs-cTnT with additional adjustments in primary prevention subjects. **Table S5A.** Major adverse cardiac event (MACE) risk according to quartiles of hs-cTnT with additional adjustments in the entire prediabetes cohort excluding those who underwent coronary revascularization within 30 days after angiography. **Table S5B.** All-cause mortality according to quartiles of hs-cTnT with additional adjustments in the entire prediabetes cohort excluding those who underwent coronary revascularization within 30 days after angiography.

## Data Availability

The authors do not have written consent from study participants to make data and materials available for public access.

## Abbreviations

CAD: Coronary artery disease
CVD: Cardiovascular disease
eGFR: Estimated glomerular filtration rate
IFG: Impaired fasting glucose
IGT: Impaired glucose tolerance
HbA1c: Glycated hemoglobin A1c
hsCRP: High-sensitivity C-reactive protein
hs-cTnT: High sensitivity cardiac troponin T
MACE: Major adverse cardiac events
OGTT: Oral glucose tolerance test
