# Supplementary material for: Prognostic value of subclinical myocardial necrosis using high-sensitivity cardiac troponin T in patients with prediabetes
Source: Cardiovasc Diabetol. 2021 Aug 21;20:171. doi: 10.1186/s12933-021-01365-9 (PMC8379781; doi:10.1186/s12933-021-01365-9)
Supplement: Supplementary file 1 — Additional file 1: Table S1. Coronary angiography findings and rates of revascularization within 30 days following procedure across hs-cTnT quartiles. Table S2A. Major adverse cardiac event (MACE) risk according to quartiles of hs-cTnT with additional adjustments in the entire prediabetes cohort. Table S2B. All-cause mortality according to quartiles of hs-cTnT with additional adjustments in the entire prediabetes cohort. Table S3A. Major adverse cardiac event (MACE) risk according to quartiles of hs-cTnT with additional adjustments in secondary prevention subjects. Table S3B. All-cause mortality according to quartiles of hs-cTnT with additional adjustments in secondary prevention subjects. Table S4A. Major adverse cardiac event (MACE) risk according to pooled quartiles 1, 2 and 3 vs. quartile 4 of hs-cTnT with additional adjustments in primary prevention subjects. Table S4B. All-cause mortality risk according to pooled quartiles 1, 2 and 3 vs. quartile 4 of hs-cTnT with additional adjustments in primary prevention subjects. Table S5A. Major adverse cardiac event (MACE) risk according to quartiles of hs-cTnT with additional adjustments in the entire prediabetes cohort excluding those who underwent coronary revascularization within 30 days after angiography. Table S5B. All-cause mortality according to quartiles of hs-cTnT with additional adjustments in the entire prediabetes cohort excluding those who underwent coronary revascularization within 30 days after angiography. [file 12933_2021_1365_MOESM1_ESM.docx]

**Table S1. Coronary angiography findings and** **rates of revascularization within 30 days following procedure across hs-cTnT quartiles**

| Angiographic findings | Total (n=2,631) | hs-cTnT  Q1 (n=658) | hs-cTnT  Q2 (n=653) | hs-cTnT  Q3 (n=661) | hs-cTnT  Q4 (n=659) | p for trend |
| --- | --- | --- | --- | --- | --- | --- |
| hs-cTnT (ng/L) |  | <8.2 | 8.2-13 | 13-21.6 | ≥21.6 |  |
| Stenosis ≥50% at any vessel (%) | 71 | 58.8 | 67.2 | 75.6 | 82.2 | <0.001 |
| Extent of coronary artery disease | | | | | | |
| 0-vessel (%) | 29.1 | 41.3 | 32.8 | 24.4 | 18.1 | <0.001 |
| 1-vessel (%) | 19.1 | 20.1 | 17.8 | 19.5 | 19.0 | 0.823 |
| 2-vessel (%) | 19.8 | 16.9 | 19.3 | 18 | 24.9 | 0.001 |
| 3-vessel (%) | 32 | 21.7 | 30.2 | 38.1 | 38.1 | <0.001 |
| Revascularization within 30 days (%) | 15.5 | 9.9 | 12.9 | 17.7 | 21.5 | <0.001 |

Abbreviations: hs-cTnT, high-sensitivity cardiac troponin T, Q, quartile

**Table S2A. Major adverse cardiac event (MACE) risk according to quartiles of hs-cTnT with additional adjustments in the entire prediabetes cohort**

| All patients (n=2,631) |  | Hazard ratio for 3-year MACE (95% CI) | | |
| --- | --- | --- | --- | --- |
|  | Q1 (n=658) | Q2 (n=653) | Q3 (n=661) | Q4 (n=659) |
| hs-cTnT (ng/L) | <8.2 | 8.2-13 | 13-21.6 | ≥21.6 |
| Unadjusted | 1 | 1.23 (0.82 - 1.84) | 2.25 (1.57 - 3.22)*** | 3.63 (2.58 - 5.1)*** |
| Adjustment 1 | 1 | 1.08 (0.72 - 1.62) | 1.65 (1.14 - 2.39)** | 2.42 (1.69 - 3.46)*** |
| Adjustment 2 | 1 | 1.43 (0.88 - 2.31) | 1.58 (0.99 - 2.54) | 2.1 (1.34 - 3.32)*** |
| Adjustment 3 | 1 | 1.08 (0.72 - 1.61) | 1.59(1.1 - 2.31)* | 2.37 (1.66 - 3.39)*** |
| Adjustment 4 | 1 | 1.11 (0.74 - 1.66) | 1.74 (1.2 - 2.51)** | 2.66 (1.87 - 3.78)*** |
| Adjustment 5 | 1 | 1.06 (0.7 - 1.61) | 1.7 (1.17 - 2.49)** | 2.66 (1.86 - 3.82)*** |
| Adjustment 6 | 1 | 1.29 (0.77 - 2.17) | 1.51 (0.92 - 2.49) | 2.46 (1.53 - 3.95)*** |
|  |  |  |  |  |

Cox proportional hazards model showing the hazard ratio for 3-year MACE (death, non-fatal myocardial infarction, non-fatal stroke) in quartiles (Q) of hs-cTnT with multiple models for adjustment.

Model 1: Adjusted for age, sex, blood pressure, low-density lipoprotein (LDL) cholesterol, high-density lipoprotein (HDL) cholesterol, estimated glomerular filtration rate

Model 2: Adjusted for age, sex, blood pressure, LDL cholesterol, HDL cholesterol, high-sensitivity C-reactive protein

Model 3: Adjusted for age, sex, blood pressure, LDL cholesterol, HDL cholesterol, cardiovascular disease

Model 4: Adjusted for age, sex, blood pressure, LDL cholesterol, HDL cholesterol, use of statins or angiotensin-converting enzyme inhibitors

Model 5: Adjusted for age, sex, blood pressure, LDL cholesterol, HDL cholesterol, hemoglobin A1c

Model 6: Adjusted for age, sex, blood pressure, LDL cholesterol, HDL cholesterol, left ventricular ejection fraction

*P<0.05 **p<0.01 ***p<0.001

**Table S2B. All-cause mortality according to quartiles of hs-cTnT with additional adjustments in the entire prediabetes cohort**

| All patients (n=2,631) |  | Hazard ratio for 5-year death (95% CI) | | |
| --- | --- | --- | --- | --- |
|  | Q1 (n=658) | Q2 (n=653) | Q3 (n=661) | Q4 (n=659) |
| Hs-cTnT (ng/L) | <8.2 | 8.2-13 | 13-21.6 | ≥21.6 |
| Unadjusted | 1 | 1.88 (1.21 - 2.94)** | 3.40 (2.26 - 5.12)*** | 5.85 (3.95 - 8.64)*** |
| Adjustment 1 | 1 | 1.55 (1.00 - 2.42) | 2.19 (1.44 - 3.34)*** | 3.37 (2.25 - 5.05)*** |
| Adjustment 2 | 1 | 1.62 (0.96 - 2.75) | 2.06 (1.24 - 3.42)** | 2.93 (1.79 - 4.82)*** |
| Adjustment 3 | 1 | 1.56(1.00 - 2.43)* | 2.23 (1.46 - 3.39)*** | 3.63 (2.42 - 5.46)*** |
| Adjustment 4 | 1 | 1.59(1.02 - 2.48)* | 2.32 (1.52 - 3.52)*** | 3.82 (2.56 - 5.70)*** |
| Adjustment 5 | 1 | 1.5 (0.95 - 2.36) | 2.14 (1.39 - 3.28)*** | 3.72 (2.47 - 5.60)*** |
| Adjustment 6 | 1 | 1.4 (0.78 - 2.52) | 1.95 (1.13 - 3.36)* | 3.24 (1.92 - 5.47)*** |
|  |  |  |  |  |

Cox proportional hazards model showing the hazard ratio for 5-year death in quartiles of hs-cTnT with multiple models for adjustment.

Model 1: Adjusted for age, sex, blood pressure, low-density lipoprotein (LDL) cholesterol, high-density lipoprotein (HDL) cholesterol, estimated glomerular filtration rate

Model 2: Adjusted for age, sex, blood pressure, LDL cholesterol, HDL cholesterol, high-sensitivity C-reactive protein

Model 3: Adjusted for age, sex, blood pressure, LDL cholesterol, HDL cholesterol, cardiovascular disease

Model 4: Adjusted for age, sex, blood pressure, LDL cholesterol, HDL cholesterol, use of statins or angiotensin-converting enzyme inhibitors

Model 5: Adjusted for age, sex, blood pressure, LDL cholesterol, HDL cholesterol, hemoglobin A1c

Model 6: Adjusted for age, sex, blood pressure, LDL cholesterol, HDL cholesterol, left ventricular ejection fraction

*P<0.05 **p<0.01 ***p<0.001

**Table S3A. Major adverse cardiac event (MACE) risk according to quartiles of hs-cTnT with additional adjustments in secondary prevention subjects**

| Secondary prevention (n=2,074) |  | Hazard ratio for 3-year MACE (95% CI) | | |
| --- | --- | --- | --- | --- |
|  | Q1 (n=519) | Q2 (n=518) | Q3 (n=518) | Q4 (n=519) |
| hs-cTnT (ng/L) | <8.8 | 8.8-14.2 | 14.2-25.2 | ≥25.2 |
| Unadjusted | 1 | 1.25 (0.85 - 1.85) | 2.07 (1.45 - 2.96)*** | 2.72 (1.92 - 3.84)*** |
| Adjustment 1 | 1 | 1.07 (0.72 - 1.58) | 1.5(1.04 - 2.16)* | 1.86 (1.31 - 2.66)*** |
| Adjustment 2 | 1 | 1.43 (0.89 - 2.29) | 1.5 (0.94 - 2.41) | 1.76(1.1 - 2.82)* |
| Adjustment 3 | 1 | 1.09 (0.74 - 1.61) | 1.58(1.1 - 2.28)* | 2.03 (1.43 - 2.88)*** |
| Adjustment 4 | 1 | 1.04 (0.7 - 1.56) | 1.56(1.08 - 2.27)* | 2.11 (1.47 - 3.02)*** |
| Adjustment 5 | 1 | 1.21 (0.73 - 2.02) | 1.39 (0.86 - 2.26) | 1.97 (1.22 - 3.16)** |
|  |  |  |  |  |

Cox proportional hazards model showing the hazard ratio for 3-year MACE (death, non-fatal myocardial infarction, non-fatal stroke) in quartiles (Q) of hs-cTnT with multiple models for adjustment.

Model 1: Adjusted for age, sex, blood pressure, low-density lipoprotein (LDL) cholesterol, high-density lipoprotein (HDL) cholesterol, estimated glomerular filtration rate

Model 2: Adjusted for age, sex, blood pressure, LDL cholesterol, HDL cholesterol, high-sensitivity C-reactive protein

Model 3: Adjusted for age, sex, blood pressure, LDL cholesterol, HDL cholesterol, use of statins or angiotensin-converting enzyme inhibitors

Model 4: Adjusted for age, sex, blood pressure, LDL cholesterol, HDL cholesterol, hemoglobin A1c

Model 5: Adjusted for age, sex, blood pressure, LDL cholesterol, HDL cholesterol, left ventricular ejection fraction

*P<0.05 **p<0.01 ***p<0.001

**Table S3B. All-cause mortality according to quartiles of hs-cTnT with additional adjustments in secondary prevention subjects**

| Secondary prevention (n=2,074) |  | Hazard ratio for 5-year death (95% CI) | | |
| --- | --- | --- | --- | --- |
|  | Q1 (n=519) | Q2 (n=518) | Q3 (n=518) | Q4 (n=519) |
| Hs-cTnT (ng/L) | <8.8 | 8.8-14.2 | 14.2-25.2 | ≥25.2 |
| Unadjusted | 1 | 2.02 (1.31 - 3.14)** | 3.25 (2.15 - 4.90)*** | 4.53 (3.04 - 6.75)*** |
| Adjustment 1 | 1 | 1.60 (1.03 - 2.48)* | 2.04 (1.34 - 3.10)*** | 2.7 (1.79 - 4.08)*** |
| Adjustment 2 | 1 | 1.59 (0.94 - 2.67) | 1.90 (1.14 - 3.14)* | 2.31 (1.38 - 3.86)*** |
| Adjustment 3 | 1 | 1.64(1.06 - 2.54)* | 2.19 (1.44 - 3.32)*** | 3.10 (2.06 - 4.65)*** |
| Adjustment 4 | 1 | 1.55 (0.99 - 2.43) | 2.05 (1.34 - 3.13)*** | 3.10 (2.05 - 4.70)*** |
| Adjustment 5 | 1 | 1.48 (0.82 - 2.67) | 2.16 (1.25 - 3.73)** | 2.87 (1.65 - 4.99)*** |
|  |  |  |  |  |

Cox proportional hazards model showing the hazard ratio for 5-year death in quartiles of hs-cTnT with multiple models for adjustment.

Model 1: Adjusted for age, sex, blood pressure, low-density lipoprotein (LDL) cholesterol, high-density lipoprotein (HDL) cholesterol, estimated glomerular filtration rate

Model 2: Adjusted for age, sex, blood pressure, LDL cholesterol, HDL cholesterol, high-sensitivity C-reactive protein

Model 3: Adjusted for age, sex, blood pressure, LDL cholesterol, HDL cholesterol, use of statins or angiotensin-converting enzyme inhibitors

Model 4: Adjusted for age, sex, blood pressure, LDL cholesterol, HDL cholesterol, hemoglobin A1c

Model 5: Adjusted for age, sex, blood pressure, LDL cholesterol, HDL cholesterol, left ventricular ejection fraction

*P<0.05 **p<0.01 ***p<0.001

**Table S4A. Major adverse cardiac event (MACE) risk according to pooled quartiles 1,2 and 3 vs. quartile 4 of hs-cTnT with additional adjustments in primary prevention subjects**

| Primary prevention (n=557) | | Hazard ratio for 3-year MACE (95% CI) |
| --- | --- | --- |
|  | Low (Q1/2/3) (n=416) | High (Q4)  (n=141) |
| hs-cTnT (ng/L)  Unadjusted | <14.4  1 | ≥14.4  8.00 (3.53 - 18.1)*** |
| Adjustment 1 | 1 | 6.57 (2.84 - 15.24)*** |
| Adjustment 2 | 1 | 2.67(1.03 - 6.92)* |
| Adjustment 3 | 1 | 6.74 (2.80 - 16.26)*** |
| Adjustment 4 | 1 | 5.97 (2.50 - 14.29)*** |
| Adjustment 5 | 1 | 2.91(1.10 - 7.69)* |
|  |  |  |

Cox proportional hazards model showing the hazard ratio for 3-year MACE (death, non-fatal myocardial infarction, non-fatal stroke) in quartile (Q) 4 compared to pooled quartiles 1,2 and 3 of hs-cTnT with multiple models for adjustment.

Model 1: Adjusted for age, sex, blood pressure, low-density lipoprotein (LDL) cholesterol, high-density lipoprotein (HDL) cholesterol, estimated glomerular filtration rate

Model 2: Adjusted for age, sex, blood pressure, LDL cholesterol, HDL cholesterol, high-sensitivity C-reactive protein

Model 3: Adjusted for age, sex, blood pressure, LDL cholesterol, HDL cholesterol, use of statins or angiotensin-converting enzyme inhibitors

Model 4: Adjusted for age, sex, blood pressure, LDL cholesterol, HDL cholesterol, hemoglobin A1c

Model 5: Adjusted for age, sex, blood pressure, LDL cholesterol, HDL cholesterol, left ventricular ejection fraction

*P<0.05 **p<0.01 ***p<0.001

**Table S4B. All-cause mortality risk according to pooled quartiles 1,2 and 3 vs. quartile 4 of hs-cTnT with additional adjustments in primary prevention subjects**

| Primary Prevention (n=557) | | Hazard ratio for 5-year death (95% CI) |
| --- | --- | --- |
| hs-cTnT (ng/L) | Low (Q1/2/3) (n=416)  <14.4 | High (Q4)  (n=141)  ≥14.4 |
| Unadjusted | 1 | 7.25 (3.58 - 14.68)*** |
| Adjustment 1 | 1 | 5.51 (2.63 - 11.56)*** |
| Adjustment 2 | 1 | 4.53 (1.77 - 11.57)** |
| Adjustment 4 | 1 | 5.46 (2.62 - 11.35)*** |
| Adjustment 5 | 1 | 5.03 (2.38 - 10.63)*** |
| Adjustment 6 | 1 | 4.36 (1.67 - 11.39)** |

Cox proportional hazards model showing the hazard ratio for 5-year death in quartile (Q) 4 compared to pooled quartiles 1,2 and 3 of hs-cTnT with multiple models for adjustment.

Model 1: Adjusted for age, sex, blood pressure, low-density lipoprotein (LDL) cholesterol, high-density lipoprotein (HDL) cholesterol, estimated glomerular filtration rate

Model 2: Adjusted for age, sex, blood pressure, LDL cholesterol, HDL cholesterol, high-sensitivity C-reactive protein

Model 3: Adjusted for age, sex, blood pressure, LDL cholesterol, HDL cholesterol, use of statins or angiotensin-converting enzyme inhibitors

Model 4: Adjusted for age, sex, blood pressure, LDL cholesterol, HDL cholesterol, hemoglobin A1c

Model 5: Adjusted for age, sex, blood pressure, LDL cholesterol, HDL cholesterol, left ventricular ejection fraction

*P<0.05 **p<0.01 ***p<0.001

**Table S5A. Major adverse cardiac event (MACE) risk according to quartiles of hs-cTnT with additional adjustments in the entire prediabetes cohort excluding those who underwent coronary revascularization within 30 days after angiography**

| All patients (n=2,223) |  | Hazard ratio for 3-year MACE (95% CI) | | |
| --- | --- | --- | --- | --- |
|  | Q1 (n=556) | Q2 (n=555) | Q3 (n=556) | Q4 (n=556) |
| hs-cTnT (ng/L) | <7.8 | 7.8-12.5 | 12.5-20.5 | ≥20.5 |
| Unadjusted | 1 | 1.00 (0.63 - 1.60) | 2.29 (1.54 - 3.41)*** | 3.36 (2.30 - 4.92)*** |
| Adjustment 1 | 1 | 0.88 (0.55 - 1.41) | 1.71 (1.14 - 2.57)** | 2.17 (1.45 - 3.24)*** |
| Adjustment 2 | 1 | 1.17 (0.68 - 2.01) | 1.47 (0.88 - 2.46) | 1.68 (1.03 - 2.74)* |
| Adjustment 3 | 1 | 0.88 (0.56 - 1.41) | 1.66 (1.1 - 2.49)* | 2.13 (1.42 - 3.19)*** |
| Adjustment 4 | 1 | 0.90 (0.57 - 1.44) | 1.79 (1.19 - 2.68)** | 2.39 (1.61 - 3.54)*** |
| Adjustment 5 | 1 | 0.89 (0.55 - 1.43) | 1.80 (1.19 - 2.72)** | 2.45 (1.64 - 3.67)*** |
| Adjustment 6 | 1 | 1.03 (0.56 - 1.89) | 1.58 (0.91 - 2.74) | 2.34 (1.38 - 3.97)** |
| Adjustment 7 | 1 | 0.89 (0.56 - 1.41) | 1.74 (1.16 - 2.61)** | 2.28 (1.53 - 3.39)*** |
| Adjustment 8 | 1 | 0.88 (0.55 - 1.41) | 1.72 (1.14 - 2.58)** | 2.27 (1.53 - 3.37)*** |
|  |  |  |  |  |

Cox proportional hazards model showing the hazard ratio for 3-year MACE (death, non-fatal myocardial infarction, non-fatal stroke) in quartiles (Q) of hs-cTnT with multiple models for adjustment.

Model 1: Adjusted for age, sex, blood pressure, low-density lipoprotein (LDL) cholesterol, high-density lipoprotein (HDL) cholesterol, estimated glomerular filtration rate

Model 2: Adjusted for age, sex, blood pressure, LDL cholesterol, HDL cholesterol, high-sensitivity C-reactive protein

Model 3: Adjusted for age, sex, blood pressure, LDL cholesterol, HDL cholesterol, cardiovascular disease

Model 4: Adjusted for age, sex, blood pressure, LDL cholesterol, HDL cholesterol, use of statins or angiotensin-converting enzyme inhibitors

Model 5: Adjusted for age, sex, blood pressure, LDL cholesterol, HDL cholesterol, hemoglobin A1c

Model 6: Adjusted for age, sex, blood pressure, LDL cholesterol, HDL cholesterol, left ventricular ejection fraction

Model 7: Adjusted for age, sex, blood pressure, LDL cholesterol, HDL cholesterol, maximal stenosis ≥50% for any vessel

Model 8: Adjusted for age, sex, blood pressure, LDL cholesterol, HDL cholesterol, extend of disease

*P<0.05 **p<0.01 ***p<0.001

**Table S5B. All-cause mortality according to quartiles of hs-cTnT with additional adjustments in the entire prediabetes cohort excluding those who underwent coronary revascularization within 30 days after angiography**

| All patients (n=2,631) |  | Hazard ratio for 5-year death (95% CI) | | |
| --- | --- | --- | --- | --- |
|  | Q1 (n=556) | Q2 (n=555) | Q3 (n=556) | Q4 (n=556) |
| Hs-cTnT (ng/L) | <7.8 | 7.8-12.5 | 12.5-20.5 | ≥20.5 |
| Unadjusted | 1 | 1.46 (0.88 - 2.42) | 3.43 (2.19 - 5.37)*** | 5.76 (3.75 - 8.84)*** |
| Adjustment 1 | 1 | 1.20 (0.72 – 2.00) | 2.23 (1.40 - 3.55)*** | 3.22 (2.06 - 5.04)*** |
| Adjustment 2 | 1 | 1.41 (0.77 - 2.56) | 2.10 (1.20 - 3.68)** | 2.87 (1.65 - 4.99)*** |
| Adjustment 3 | 1 | 1.21 (0.73 - 2.02) | 2.29 (1.44 - 3.65)*** | 3.50 (2.22 - 5.51)*** |
| Adjustment 4 | 1 | 1.24 (0.74 - 2.07) | 2.38 (1.50 - 3.77)*** | 3.69 (2.36 - 5.75)*** |
| Adjustment 5 | 1 | 1.21 (0.72 - 2.04) | 2.20 (1.37 - 3.54)*** | 3.67 (2.33 - 5.79)*** |
| Adjustment 6 | 1 | 1.04 (0.51 - 2.09) | 1.98 (1.07 - 3.67)* | 3.42 (1.89 - 6.19)*** |
| Adjustment 7 | 1 | 1.21 (0.73 - 2.02) | 2.32 (1.46 - 3.69)*** | 3.56 (2.27 - 5.58)*** |
| Adjustment 8 | 1 | 1.21 (0.72 - 2.01) | 2.28 (1.44 - 3.62)*** | 3.5 (2.24 - 5.47)*** |
|  |  |  |  |  |

Cox proportional hazards model showing the hazard ratio for 5-year death in quartiles of hs-cTnT with multiple models for adjustment.

Model 1: Adjusted for age, sex, blood pressure, low-density lipoprotein (LDL) cholesterol, high-density lipoprotein (HDL) cholesterol, estimated glomerular filtration rate

Model 2: Adjusted for age, sex, blood pressure, LDL cholesterol, HDL cholesterol, high-sensitivity C-reactive protein

Model 3: Adjusted for age, sex, blood pressure, LDL cholesterol, HDL cholesterol, cardiovascular disease

Model 4: Adjusted for age, sex, blood pressure, LDL cholesterol, HDL cholesterol, use of statins or angiotensin-converting enzyme inhibitors

Model 5: Adjusted for age, sex, blood pressure, LDL cholesterol, HDL cholesterol, hemoglobin A1c

Model 6: Adjusted for age, sex, blood pressure, LDL cholesterol, HDL cholesterol, left ventricular ejection fraction

Model 7: Adjusted for age, sex, blood pressure, LDL cholesterol, HDL cholesterol, maximal stenosis ≥50% for any vessel

Model 8: Adjusted for age, sex, blood pressure, LDL cholesterol, HDL cholesterol, extend of disease

*P<0.05 **p<0.01 ***p<0.001
